# Sex-Specific Impact of Metabolic Syndrome on Brain Structures Vulnerable to Alzheimer’s Disease: A Cross-Sectional Study in a Brazilian Cohort

**DOI:** 10.3390/brainsci15121341

**Published:** 2025-12-17

**Authors:** Rodrigo Hohl, Fernanda Gabriele Fernandes de Morais, Tâmara Pessanha Taporoski, André Brooking Negrão, Simon L. Evans, Camila Maciel de Oliveira, Alexandre da Costa Pereira, Rafael de Oliveira Alvim

**Affiliations:** 1 Department of Biophysics and Physiology, Institute of Biological Sciences, Federal University of Juiz de Fora (UFJF), Campus Universitário, Rua José Lourenço Kelmer, s/n—São Pedro, Juiz de Fora 36036-900, MG, Brazil; 2 Department of Psychiatry, University of Wisconsin-Madison, Madison, WI 53705, USA; 3 Laboratory of Genetics and Molecular Cardiology, Instituto do Coração (Heart Institute), University of São Paulo, São Paulo 05508-010, SP, Brazil; 4 Faculty of Health and Medical Sciences, University of Surrey, Guildford GU2 7YH, UK; 5 Department of Physiological Sciences, Federal University of Amazonas (UFAM), Manaus 69067-005, AM, Brazil

**Keywords:** dementia, aging, temporal lobe, gray matter, sex-related stress

## Abstract

**Background:** Metabolic syndrome (MetS) is linked to brain degeneration and Alzheimer’s disease (AD). Women, especially during menopausal transition, show increased susceptibility to AD-related brain changes. This study investigated the sex-specific neurostructural impact of MetS on brain regions vulnerable to AD. **Methods:** This cross-sectional study analyzed data from 500 participants (303 women, 197 men) from the Baependi Heart Study cohort, Brazil. High-resolution T1-weighted MRI scans were used for volumetric analysis of AD-related regions of interest (ROIs). Non-parametric quantile regression models compared ROI volumes between MetS and Non-MetS groups, stratified by sex and age (median split), adjusting for age and education. **Results:** No significant differences in ROI volume were observed between the MetS and Non-MetS groups in men. In women, findings were age-dependent. The younger cohort (≤48 years) with MetS exhibited significantly smaller left hippocampal volume (*p* = 0.02) and a trend toward smaller left middle temporal gyrus volume (*p* = 0.05) compared to Non-MetS. The older cohort (>48 years) with MetS showed a significantly larger right amygdala volume (*p* < 0.001). Furthermore, age-related volume decline in the hippocampus and middle temporal gyrus was significant in Non-MetS women but not in women with MetS, suggesting that MetS may be a confounding factor in age-related neurodegeneration. **Conclusions:** MetS is associated with sex-specific alterations in AD-vulnerable brain structures. In women, MetS may influence medial temporal lobe atrophy pre-menopause, and is linked to amygdala enlargement post-menopause. These exploratory results generate the hypothesis that MetS may uniquely predispose women to AD-related neurodegeneration, which requires critical longitudinal confirmation.

## 1. Introduction

Metabolic syndrome (MetS) is a complex medical condition characterized by key metabolic risk factors, including abdominal obesity, insulin resistance, high blood pressure, and abnormal cholesterol levels. In Brazil, national health survey data estimate a prevalence of 38.4% for MetS [1]. Among its components, a high waist circumference (65.5%) and low HDL cholesterol (49.4%) are the most common, with particular concern regarding their high rates among young adults [1]. These data highlight the urgent need for a thorough understanding of MetS in the Brazilian population, considering its widespread prevalence and increasing trend.

While traditionally linked to cardiovascular outcomes, MetS has also been associated with significant structural and functional brain changes, thereby impairing cognitive functions such as attention, memory, and executive functions [2]. Evidence indicates MetS accelerates the degeneration of both white and gray matter, affecting brain regions essential for cognitive and motor abilities [3]. This accelerated brain degeneration may, in turn, be a key factor in the progression of Alzheimer’s disease (AD) and other neurodegenerative disorders [4].

In the neurodegenerative landscape of AD, many brain regions are affected. The hippocampus is among the first structures to undergo atrophy. Its integrity is crucial for episodic memory and plays a key role in the progression of mild cognitive impairment to AD [5]. The entorhinal cortex and the amygdala are also part of the medial temporal memory circuit and are impacted in the early stages of AD. Their functions include forming new memories, often accompanied by significant neuronal loss [6,7,8].

Beyond these traditionally studied regions, structures such as the middle temporal gyrus and the right precuneus have shown volumetric changes associated with AD progression. These areas, which are vital to the associative cortex and cognitive functions, also exhibit reductions as part of the broader spectrum of volumetric alterations in AD [7,9,10,11]. Therefore, selecting these regions of interest (ROIs) is justified given their functional importance and early susceptibility to AD, which may be further exacerbated by MetS-related atrophy.

Women constitute two-thirds of all individuals affected by AD worldwide [12]. This sex disparity extends beyond differences in lifespan, emphasizing the urgent need to explore sex-specific vulnerabilities in brain aging and neurodegeneration. Research indicates that the menopausal transition—a period of significant endocrine change during midlife—is a critical window of increased vulnerability for women [13,14], during which MetS may serve as a key modulator, substantially elevating the risk of AD after menopause. Additionally, the variation in dementia risk among women globally highlights the strong influence of socio-cultural factors such as disparities in education and economic opportunities, demonstrating a complex interplay of biological and environmental influences [15].

Considering the widespread prevalence of MetS and its links to brain degeneration and AD, research must address known sex- and age-dependent differences in vulnerability. This is especially important for women during menopause and across different populations and cultural settings [16]. Thus, this study aims to explore the neurostructural effects of MetS in men and women by focusing on specific ROIs involved in AD progression and accounting for the interplay among age, education, and sex. We also seek to contribute to a broader, multicultural understanding of AD risk factors, which could enable tailored interventions for individuals at risk. Given the cross-sectional design and the cohort’s non-demented status, the aim of this study is not to provide diagnostic prediction or clinical screening, but rather to use statistical modeling to identify novel sex- and age-specific structural brain associations with metabolic syndrome. The results generated here are intended to serve as mechanistic hypotheses that guide the design and focus of future longitudinal and epidemiological studies aimed at confirming the causality and progression of MetS-related neurodegeneration.

## 2. Methods

### 2.1. Study Population

Participants for this cross-sectional and exploratory analysis were recruited from the Baependi Heart Study. Established in 2005, this ongoing Brazilian epidemiological cohort uses a longitudinal, family-based design to assess environmental and genetic influences on cardiovascular disease risk factors [17]. Baependi is a town in a rural area in southeastern Brazil, known for its limited inbound migration, cohesive culture, and high admixture of European, African, and Native American ancestries. A notable strength of this dataset is the wide range of education levels among the population. The methods for participant recruitment and characteristics of the study population have been described in detail previously [18].

This study presents a cross-sectional analysis of data from 500 participants, selected from a larger pool of 1691 individuals during the project’s second collection wave (2010–2015). Due to resource constraints, MRI was performed only on a subset of the total cohort. Initial screening for participation required subjects to meet general eligibility criteria. Individuals were excluded based on pre-existing brain pathologies (e.g., dementia, tumors, stroke) or standard MRI contraindications (e.g., claustrophobia, metal implants). The remaining subjects were contacted for an additional MRI scan. Financial constraints limited the MRI procedure to 550 scans, and 543 participants participated. The further selection process for this study required participants to have a complete, artifact-free MRI scan, with image quality confirmed by expert evaluation, and to be free of diabetes. All procedures were conducted in accordance with the Declaration of Helsinki, and the protocol was approved by the local ethics committee (Hospital das Clinicas, University of São Paulo, Brazil, number 0494/10, approval date 27 October 2010). All participants provided written informed consent. This MRI dataset has also served as the basis for prior published analyses [19,20].

### 2.2. Measures

Physical measurements included waist circumference (cm) and blood pressure. Using a standard digital sphygmomanometer (OMRON, Kyoto, Japan), blood pressure was measured on the left arm of seated participants after a 5 min rest period. The final systolic (SBP) and diastolic (DBP) values were determined by averaging three separate readings, each taken at least three minutes apart. For biochemical analyses, blood samples were obtained after a 12 h fast. Standard laboratory techniques were used to evaluate levels of high-density lipoproteins (HDL), triglycerides, and fasting glucose. Glycated hemoglobin (HbA1c) levels were quantified using high-performance liquid chromatography (HPLC).

### 2.3. Definition of MetS

Metabolic syndrome was diagnosed according to the JIS (Joint Interim Statement) criteria [21], which requires the presence of any three of the following five risk factors: blood pressure (systolic blood pressure ≥130 mmHg and/or diastolic blood pressure ≥ 85 mmHg, or anti-hypertensive drug treatment); fasting glucose (≥100 mg/dL, or anti-diabetic drug treatment); HDL-c (<40 mg/dL for men and <50 mg/dL for women, or hypolipidemic drug treatment); triglycerides (≥150 mg/dL, or hypolipidemic drug treatment); and waist circumference (≥90 cm in men and ≥80 cm in women). Fifty-five (*n* = 56) participants diagnosed with MetS were under hypolipidemic drug treatment, thirty-eight (*n* = 38) under anti-diabetic drug treatment, and eight (*n* = 8) under anti-hypertensive drug treatment.

Based on the JIS criteria, participants were categorized into groups defined by the presence or absence of the condition: those diagnosed with metabolic syndrome were defined as the MetS group, and those who did not meet the criteria were defined as the Non-MetS group. The term “control group” is used throughout this manuscript to provide a clear, immediately distinguishable contrast for readability. This cohort ultimately comprised 197 men (MetS, *n* = 82; Non-MetS, *n* = 115) and 303 women (MetS, *n* = 151; Non-MetS, *n* = 152).

### 2.4. MRI Acquisition

As described elsewhere [19], MRI scans were obtained at the Hospital Cônego Monte Raso in Baependi on a 1.5 T MAGNETOM (Siemens, Munich, Germany). High-resolution T1 images were acquired using a three-dimensional fast spoiled gradient echo T1-weighted sequence (Voxel size 1 mm^3^, 160 slices, Matrix Size 256 × 256, TR 1700 ms, TE 5.1 ms, flip angle 120, inversion time 850 ms). A T2-weighted FLAIR sequence (Matrix Size 280 × 320, Voxel size 0.719 × 0.719 × 6.32 mm, axial slice width 5.5 mm with 0.82 mm interslice gap, 20 slices, TR 1000.2 ms, TE 109.3 ms, flip angle 150, inversion time 2500 ms) was acquired.

### 2.5. Volumetric Analyses

All raw images were visually inspected for quality. Cortical reconstruction and volumetric segmentation were performed using the Freesurfer 6.0.0 image analysis suite (http://surfer.nmr.mgh.harvard.edu, accessed on 1 November 2025). The reconstruction pipeline employed by Freesurfer, which includes intensity normalization, motion correction, and the exclusion of non-brain tissue, was implemented using a hybrid watershed/surface-deformation approach. The images are transformed to Talairach space. The FreeSurfer reconstructions were checked visually for errors. Cortical volume estimates were calculated based on the Destrieux atlas [22]. In total, volume estimates for 235 (subcortical and cortical) regions were extracted, and five AD-related ROIs from the right and left hemispheres were included in the analyses (e.g., hippocampus, entorhinal cortex, middle temporal gyrus, precuneus, and amygdala). To account for differences in head size, the volume of each ROI was divided by the participant’s estimated total intracranial volume (ICV).

### 2.6. Statistical Analysis

The Shapiro–Wilk test was used to assess normality, and Levene’s test was used to assess homogeneity of variances. The normality assumption was violated for all ROIs, clinical characteristics, age, and years of education. Therefore, statistical analyses were performed using quantile regression, which allows investigation of the relationship between predictor variables across different quantiles of the dependent variable’s distribution and is robust to deviations from normality and the presence of outliers.

Quantile regression (QR) analyses were the primary statistical approach, justified by the violation of the normality assumption observed in all ROIs. QR is robust to non-normal distributions and heterogeneity of variance, providing a reliable method to model the relationship between predictors and the dependent variable across various quantiles. To account for sex differences, these analyses were stratified by sex; that is, separate models were constructed for men and women. For this study, the focus was explicitly on the 0.50 quantile (Q2, the median). The core quantile regression model included metabolic syndrome status (categorized as MetS or Non-MetS) as the primary factor of interest. To control for potential confounding factors, the model was adjusted for age in years and years of education. Within this framework, the *p*-value associated with the coefficient for metabolic syndrome status directly indicated the statistical significance of the difference in the conditional median of the respective brain volume between the MetS and Non-MetS groups, after accounting for the influence of age and years of education.

To visualize the inferential effect sizes of metabolic syndrome on regional brain volumes, a coefficient plot was constructed based on the QR results (Q2). Point estimates in the figure represent the beta coefficients (βMetS) adjusted for covariates. The 95% confidence intervals (95% CIs) were calculated using the standard errors (SEs) derived from the model, with the standard formula: 95% CI = β ± (1.96 × SE).

**Figure 1:**
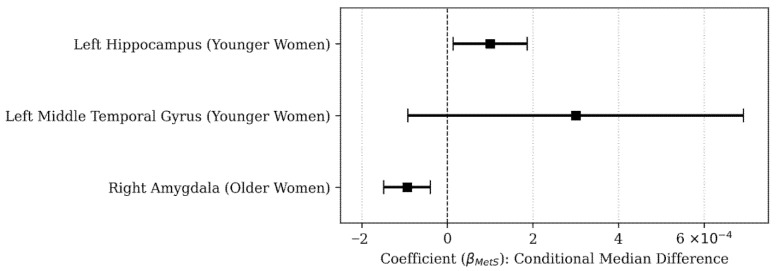
Quantile regression estimates (Q2) for the association between metabolic syndrome and volumes of regions of interest. Black squares represent beta coefficients, indicating the conditional median difference in volume (normalized by total intracranial volume) between groups. Horizontal bars indicate 95% confidence intervals (95% CIs). Models adjusted for age and education.

Furthermore, an additional model was employed to investigate whether the effect of MetS on brain volume was age-dependent. This model included an interaction term between MetS status and age across the entire age range for both men and women. This approach was based on the biological rationale that MetS’s effects on the brain may be modulated by age, particularly in the context of post-menopausal hormonal changes. This second modeling approach is shown in Figure 1.

Data are presented as the median (Q2) and interquartile ranges (Q1–Q3). The nonparametric Mann–Whitney U test was used to compare metabolic syndrome profiles, age, education, and intracranial volume between the MetS and Non-MetS groups in men and women. The one-sample Wilcoxon signed-rank test was used to compare the MetS groups of both sexes with the JIS reference values for MetS diagnosis. Statistical significance was defined as *p* < 0.05. Descriptive and inferential statistics were analyzed using IBM SPSS Statistics version 27.

## 3. Results

Table 1 summarizes the clinical and demographic characteristics of the participants, stratified by sex and MetS status. Participants with MetS were significantly older (*p* < 0.001) than those in their respective Non-MetS groups. Across both sexes, the MetS groups also exhibited a considerably less favorable metabolic syndrome profile compared to their Non-MetS counterparts (*p* < 0.001 for all variables). Additionally, the median education level was significantly lower among women with MetS (6 years (4–11)) than among Non-MetS women (11 years (4–12)) (*p* < 0.001). The intracranial volume (ICV) did not show statistically significant differences between the MetS and Non-MetS groups in either sex.

Comparisons were made between the MetS groups (both men and women) and the JIS reference criteria to confirm that individuals categorized as having MetS met the diagnostic thresholds. Participants in the Non-MetS group did not meet the JIS criteria for MetS. When comparing JIS values by MetS groups, fasting glucose was significantly below the 100 mg/dL threshold in both sexes (*p* < 0.001). Hemoglobin A1c (HbA1c) followed the same pattern, being significantly lower than the normal reference value of 5.7%. Conversely, waist circumference and triglyceride levels were significantly above the reference values, while HDL cholesterol was significantly below the reference values in both sexes (*p* < 0.001). Diastolic blood pressure was below the reference values for both sexes (*p* < 0.001). Regarding systolic blood pressure, women’s values were lower than 130 mmHg (*p* < 0.001), whereas men’s values did not differ significantly from this threshold.

### 3.1. Volumetric Analysis of ROIs by Metabolic Syndrome Status and Age Subgroups

Table 2 and Table 3 present the median relative volumes of ROIs in participants below and above the 50% age percentile, stratified by sex and MetS status.

### 3.2. Participants Below the 50th Age Percentile (Q2)

Among men, no statistically significant differences in ROI volumes were observed between the Non-MetS and MetS groups.

For women in the younger subgroup, the MetS group was significantly older (41 years old; *p* < 0.001) than the Non-MetS group (36 years old). While most ROI volume comparisons did not show significant differences, the quantile regression model (Q2), adjusted for age and education, revealed significant findings for the medial temporal lobe. The quantile regression coefficient for metabolic syndrome status on left hippocampus volume was statistically significant (β = 0.0001, SE = 4.41× 10^−5^, *p* = 0.02). This positive coefficient indicates that the conditional median volume of the left hippocampus was significantly smaller in women with MetS compared to the Non-MetS group (Table 4 and Figure 1). Similarly, the left middle temporal gyrus showed a marginally significant difference (β = 0.0003, SE = 0.0002, *p* = 0.05). This positive coefficient indicates that the MetS group again exhibited a smaller conditional median volume for the middle temporal gyrus. For descriptive purposes, the median volume of the left hippocampus was 0.27% [0.26–0.28%] in the MetS group compared to 0.28% [0.27–0.30%] in the Non-MetS group. The median volume for the left middle temporal gyrus was 0.48% [0.43–0.53%] in the MetS group compared to 0.51% [0.48–0.57%] in the Non-MetS group.

### 3.3. Participants Above the 50th Age Percentile (Q2)

Within the older men subgroup, the MetS group had a significantly higher level of education (8 years (4–11)) than the Non-MetS group (4 years (4–10)) (*p* < 0.001). No statistically significant differences were observed between the Non-MetS and MetS groups in any of the ROIs volumes (Table 3).

For women in the older subgroup (median age > 48 years), the quantile regression coefficient for metabolic syndrome status on right amygdala volume was highly significant (β = −9.41 × 10^−5^, *p* < 0.001). This negative coefficient indicates that the conditional median volume of the right amygdala was significantly larger in women with MetS compared to the Non-MetS group (Table 4 and Figure 1). No significant differences were observed in the volumes of the other evaluated ROI in this subgroup. For descriptive purposes, the median volume of the right amygdala was 0.098% [0.089–0.105%] in the MetS group compared to 0.090% [0.083–0.101%] in the Non-MetS group.

Table 4 and Figure 1 summarize the inferential parameters for the ROI where significant or near-significant differences were observed in women.

### 3.4. Quantile Regression (q = 0.5) of Relative ROI Volume and Age (Years)

**Figure 2:**
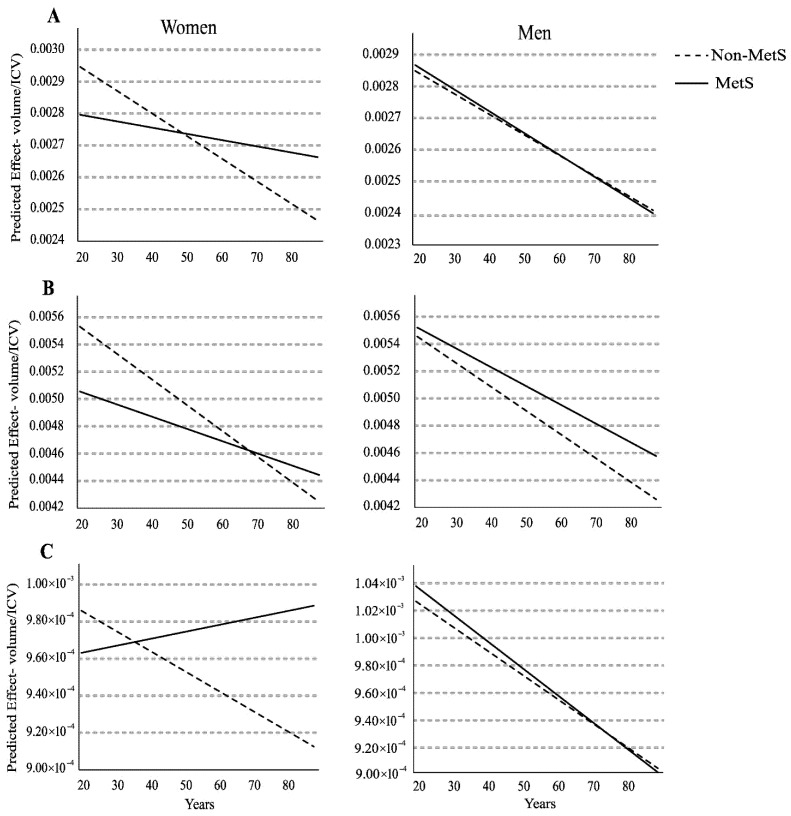
Age-related trends of predicted volumes from the quantile regression model (q = 0.5) for the left hippocampus (**A**), left middle temporal gyrus (**B**), and right amygdala (**C**). Volume normalized by ICV. Data for men (*n* = 197) and women (*n* = 303) are presented by MetS (solid line: *n*: men = 82 and women = 152) and Non-MetS (dashed line: *n*: men = 115 and women = 151) groups. Specific beta coefficients and their statistical significance are reported in the text. MetS: metabolic syndrome.

Figure 2 shows the results of the quantile regression model (Q2) for ROI volumes normalized by ICV, revealing distinct patterns of interaction with age in men (*n* = 197) and women (*n* = 303), divided into Non-MetS (*n*: men = 115 and women = 151) and MetS (*n*: men = 82 and women = 152) groups, as shown in Table 1.

### 3.5. Relative Left Hippocampus Volume

In men, a significant decline in hippocampus volume with increasing age was observed in both the Non-MetS (β = −6.18 × 10^−6^, SE = 2.20 × 10^−6^, *p* = 0.006) and the MetS groups (β = −6.58 × 10^−6^, SE = 2.90 × 10^−6^, *p* = 0.024). The decline in hippocampal volume with age was consistent and parallel across groups in males (Figure 2A). In women, age was significantly associated with a decrease in volume in the Non-MetS group (β = −7.08 × 10^−6^, SE = 1.81 × 10^−6^, *p* < 0.001). In contrast, the interaction of left hippocampus volume with age was not significant in the MetS group (β = −1.95 × 10^−6^, SE = 1.93 × 10^−6^, *p* = 0.313).

### 3.6. Relative Left Middle Temporal Gyrus Volume

For men, a significant decline in volume with increasing age (Figure 2B) was demonstrated in both the Non-MetS (β = −1.68 × 10^−5^, SE = 4.76 × 10^−6^, *p* < 0.001) and the MetS groups (β = −1.32 × 10^−5^, SE = 6.27 × 10^−6^, *p* = 0.036). For women, a significant decline in volume associated with age was observed in the Non-MetS group (β = −1.89 × 10^−5^, SE = 4.91 × 10^−6^, *p* < 0.001). The interactions volume × age was not statistically significant in the Mets group (β = −8.99 × 10^−6^, SE = 5.24 × 10^−6^, *p* = 0.087).

### 3.7. Relative Right Amygdala Volume

Age was not significantly associated with a decline in volume within men (Figure 2C) for either the Non-MetS (β = −1.72 × 10^−6^, SE = 9.47 × 10^−7^, *p* = 0.072) or the MetS groups (β = −1.91 × 10^−6^, SE = 1.25 × 10^−6^, *p* = 0.126). In women, age was also not significantly associated with changes in volume in the Non-MetS group (β = −1.08 × 10^−6^, SE = 8.95 × 10^−7^, *p* = 0.229) or the MetS groups (β = 3.74 × 10^−7^, SE = 9.55 × 10^−7^, *p* = 0.695).

### 3.8. Post Hoc Exploratory Analysis: Age Effects in Non-MetS Women in the Left Hippocampus and Middle Temporal Gyrus

To further investigate the potential influence of aging and the menopausal transition on brain structure independent of metabolic syndrome, we performed a post hoc exploratory analysis comparing specific ROI volumes between the younger (≤48 years, *n* = 113) and older (>48 years, *n* = 38) Non-MetS women, which showed a difference between the MetS and Non-MetS groups in younger ages.

We found that older Non-MetS women (post-menopausal age range) exhibited significantly lower left hippocampal volumes than younger Non-MetS women (pre-/perimenopausal age range) (Mann–Whitney U test, *p* = 0.003). The median standardized volume (in % of ICV) for younger Non-MetS women was 0.28 [Q1–Q3: 0.27–0.30] (Table 2), while for older Non-MetS women it was 0.26 [Q1–Q3: 0.25–0.29] (Table 3).

A similar significant difference was observed for the left middle temporal gyrus, with older Non-MetS women showing lower volumes than younger Non-MetS women (Mann–Whitney U test, *p* < 0.001). The median standardized volume for younger Non-MetS women was 0.51 [Q1–Q3: 0.48–0.57] (Table 2), compared to 0.47 [Q1–Q3: 0.44–0.52] for older Non-MetS women (Table 3).

## 4. Discussion

This study investigated the neurostructural association of MetS, explicitly focusing on ROIs implicated in AD progression. This research aimed to account for recognized heightened risks observed in women in perimenopause and after menopause within a South American context. Ultimately, the goal was to facilitate earlier identification of AD-related brain changes within individuals at-risk for AD. Our data show that for women, MetS was associated with significantly lower left hippocampus and a trend for the left middle temporal gyrus volumes in the 32–47 years of age range (Table 2). Interestingly, no significant interaction was observed between ROI volume and age within the women MetS group, a finding that contrasts with the expected age-related ROI volumetric decline observed in the Non-MetS group (Figure 2A,B). Furthermore, in the 56–65 age range, our data indicate a noteworthy increase in amygdala volume among women with MetS (Table 3). Conversely, for men, no significant differences were observed in any of the ROIs volumes between MetS and Non-MetS groups at any group age; however, a significant age-related decrease in left hippocampus and middle temporal gyrus volumes was found across the entire age range analyzed.

As shown in Table 1, the MetS groups in our study comprised older subjects, specifically men aged 47–66 years and women aged 48–66 years. This aligns with existing literature, which consistently shows that the prevalence of MetS increases with age in both sexes across various cultural backgrounds [23,24,25]. Notably, this prevalence rises considerably in menopausal women aged 50 through 59 years [26]. Furthermore, our MetS groups for both sexes exhibited significantly elevated waist circumference and triglycerides, while HDL cholesterol levels were significantly below the JIS reference values. These findings are in accordance with the most prevalent MetS outcomes reported in Brazilian cohorts [1]. Consistent with previous research, MetS in the Brazilian population is particularly prevalent among older women with lower educational attainment [1], a trend also evident in our data (Table 1).

According to the study by Pompei et al. (2022) [27], the median age at menopause in Brazil is 48 (45–51) years, with menstrual irregularities typically beginning around 46 (44–49) years. In this context, the quantile regression analysis below the 50th percentile (Q2) of age likely represents a group of women generally before the perimenopausal transition (Table 2). Interestingly, within this younger cohort, women in the MetS group exhibited lower left hippocampal volume and reduced left middle temporal gyrus volume compared with the Non-MetS group. In turn, this trend was not observed in men. This suggests that MetS may exert a greater influence on the volumes of these ROIs in premenopausal women than in age-matched men. Furthermore, while age-related hippocampal-medial temporal atrophy is well documented [28,29,30], a significant interaction between age and hippocampal volume decrease was observed across all groups (i.e., men: Non-MetS/MetS and women: Non-MetS) except in the MetS group of women (Figure 2A). A similar pattern was found for the left middle temporal gyrus (Figure 2B). Collectively, these results suggest that women, particularly at earlier ages, are more vulnerable to the effects of MetS on structural brain changes, and that MetS itself may act as a confounding factor in the interaction between age and atrophy of the left hippocampus and middle temporal gyrus.

Medial temporal lobe structures, including the medial occipitotemporal, middle, and inferior temporal gyri, are among the earliest neocortical sites affected in AD, with atrophy in these regions potentially signaling future AD onset in non-demented individuals [31]. Hippocampal volume measurements are also highly effective in discriminating controls from AD cases [30]. In the present study, our findings suggest that ROIs in the left temporal lobe, particularly the left hippocampus and middle temporal gyrus, may be more sensitive in discriminating non-demented MetS women from Non-MetS. This aligns with AD literature supporting greater female vulnerability to hippocampal atrophy than males [32,33].

In this study, metabolic syndrome is associated with smaller left hippocampal volume in younger women. However, the association between MetS and reduced hippocampal volume relative to Non-MetS was not observed among women at older ages (Table 3). Women demonstrate unique patterns of hippocampal atrophy closely tied to hormonal transitions, where the withdrawal of neuroprotective gonadal steroids during menopause initially triggers a phase of accelerated structural decline [34]. Longitudinal data indicate that post-menopausal women experience a significantly higher rate of hippocampal atrophy—averaging 3.3% over three years—compared to premenopausal women and men [13]. To investigate hippocampal atrophy due to aging in the absence of MetS in our dataset, we performed a post hoc exploratory analysis comparing the hippocampal volumes of young (Table 2) and older Non-MetS women (Table 3), below and above Q2, respectively. We found that Non-MetS older women had significantly lower left hippocampal volume than Non-MetS younger women, an effect we attribute to aging in addition to post-menopause. A similar trend was observed for the left middle temporal gyrus. Therefore, our finding of reduced left hippocampal volume in Non-MetS post-menopausal women follows the aging pattern. However, this accelerated loss may not be permanent; evidence suggests a subsequent stabilization in which hippocampal volume remains relatively constant in the later post-menopausal decades, specifically between ages 50 and 70 [35], as also indicated by data showing that grey matter volume loss stabilizes post-menopause [36]. This phenomenon of delayed volume preservation is hypothesized to result from compensatory adult neurogenesis observed in female rats within the subventricular zone of the dentate gyrus. This reparative mechanism may recover and reactivate years after the final menstrual period to counteract the initial impact of estrogen loss [37]. Furthermore, this stabilization aligns with recent findings that hippocampal microstructural changes follow a non-linear trajectory dependent on age and initial condition, where older adults with already high levels of pathological change show slower rates of further deterioration, suggesting a saturation effect in the degenerative process [38].

In this vein, we hypothesize that MetS is a confounding factor in the rate of age-related decline in temporal lobe ROIs volumes (Figure 2A,B) of women, and early or accelerated atrophy associated with MetS stabilizes in older ages due to a saturation effect in the neurodegeneration, thereby hindering the impact of MetS on hippocampal atrophy. Furthermore, we hypothesize that early structural reduction at younger ages could compromise the initial cognitive resilience or neural reserve observed in women, potentially negating their initial advantage compared to men [39]. Specifically, this framework posits that women typically possess an initial cognitive resilience to hippocampal atrophy, maintaining memory function despite amyloid burden, which serves as a protective buffer before clinical symptoms emerge [39]. Our findings suggest that MetS at earlier ages may increase the risk of AD pathology vulnerability in women. Supporting this, longitudinal studies have linked smaller initial left hippocampal volumes to subsequent development of dementia [40]. Furthermore, left-hemispheric volumetric measures of medial temporal lobe structures have demonstrated superior accuracy in discriminating mildly demented AD patients from controls [41].

It is crucial to interpret these findings within the specific context of the Baependi Heart Study, a rural Brazilian cohort with a distinct metabolic profile compared to many international urban populations. A recent narrative review [39] highlights diabetes and hypertension as sex-specific drivers of white matter integrity loss and brain atrophy in women. However, our cohort presents a unique phenotype: women in the MetS group exhibited fasting glucose, HbA1c, and blood pressure levels that, on average, remained below clinical thresholds. Instead, metabolic risk in this population was predominantly driven by abdominal obesity (waist circumference) and dyslipidemia, both of which were significantly altered. This suggests that, in this specific national context, central adiposity and lipid dysregulation are sufficient independent drivers of the observed early hippocampal atrophy in young women, even in the absence of the compounding neurotoxic effects of hyperglycemia or uncontrolled hypertension that may be observed in other cohorts [42,43].

In contrast to the findings for the left hippocampus and middle temporal gyrus, we observed that the right amygdala volume in the MetS group of older women was significantly greater than in the Non-MetS group. Similar to our findings, Opel et al. (2021) [44] reported that obesity (BMI > 30) was associated with significantly increased amygdala volumes in a cohort of 6420 participants (56.95% women). Their study further showed a more pronounced increase in right amygdala volume (Cohen’s d = 0.211) than in the left (Cohen’s d = 0.123). In addition, Zhang et al. (2018) [45] found that chronic stress can induce morphological changes in the amygdala, such as dendritic growth and increased arborization in basolateral amygdala neurons.

Considering these findings, it can be hypothesized that, in post-menopausal women in this cohort with low educational attainment and metabolic syndrome characteristics, structural growth alterations induced by inflammatory stress (e.g., synaptogenesis, dendritic arborization) could attenuate or partially counterbalance the reduction in amygdala volume, acting as a structural compensatory mechanism. An increased right amygdala volume may indicate a greater risk of brain pathology, as neuroinflammation is implicated in many neurological disorders, and the inflammation associated with metabolic syndrome increases the likelihood and severity of these conditions [46]. Moreover, larger-scale population imaging (12,087 participants: 52.8% women; mean age 62 years, range 45–76 years) found smaller cortical and many subcortical volumes with higher adiposity but noted the combined amygdala volume as an exception, reinforcing that the amygdala behaves differently from most gray-matter regions in relation to body fat [47]. However, this finding requires confirmation in longitudinal studies that control for MetS and obesity while also identifying menopausal transition periods and inflammatory markers.

The main limitation of our study is its cross-sectional design, which precludes establishing causal relationships. Consequently, our interpretations regarding the onset and mechanisms of brain structural abnormalities in MetS require validation through longitudinal research before definitive conclusions can be drawn. Furthermore, this study represents a secondary analysis of a dataset collected initially to investigate cardiovascular outcomes associated with sociodemographic status. As a brain MRI was not a primary endpoint of the original study, the available sample size for the present investigation was inherently limited.

It is also necessary to consider the selection bias in the MRI subsample, as participants were selected based on image quality and availability, which may limit generalizability to the broader cohort. An additional limitation is the lack of precise control over the peri- to post-menopausal transition, which necessitates using the 50th age percentile as the grouping variable for the analysis. However, the non-parametric nature of the data and the presence of outliers also informed the statistical approach. The application of quantile regression with a median split (Q2), partially adjusted for age, increased the likelihood that women in the upper quantile were post-menopausal. The use of quantile regression ensured our analysis of the conditional median was robust to non-normality and heterogeneity, providing the necessary statistical confidence for our exploratory findings.

## 5. Conclusions

This study provides compelling cross-sectional evidence for a sex-specific impact of MetS on brain structures vulnerable to AD. Our findings suggest that in women, MetS is associated with early atrophy in the left hippocampus and middle temporal gyrus, providing a novel cross-sectional hypothesis that MetS may accelerate the timeline of neurodegeneration in these ROIs. Conversely, in older, post-menopausal women, MetS was linked to an enlarged right amygdala, which may reflect a complex neuroinflammatory response rather than a neuroprotective effect. These significant structural alterations were notably absent in men, highlighting that women are uniquely susceptible to the neurostructural consequences of MetS. The observation that older MetS women lack a significant volume difference relative to the Non-MetS group—which we hypothesize is due to a MetS confounding factor in age-related atrophy and saturation of hippocampal neurodegeneration—does not negate the relevance of the findings in the young cohort. Instead, this complex pattern serves as a critical hypothesis for longitudinal confirmation, indicating that the window of MetS vulnerability precedes the post-menopausal period. Taken together, these results underscore MetS as a crucial early risk factor that may place women on a trajectory toward heightened AD vulnerability. We emphasize that these associations are not intended for individual AD prediction but are valuable for identifying high-risk biological pathways to guide preventative research. Future longitudinal studies are essential to confirm these findings and elucidate the underlying mechanisms. Specifically, investigations should focus on the interaction between MetS and known markers of AD vulnerability, such as Apolipoprotein E (APOE) status, alongside ROI volumes. Such data are necessary to contextualize MetS’s impact on neurodegenerative risk, ultimately informing the development of targeted preventive strategies for this at-risk population.

## Figures and Tables

**Table 1:** Demographic and clinical characteristics of participants, stratified by sex and metabolic syndrome status.

	Men		Women	
	Non-MetS (*n* = 115)	MetS (*n* = 82)	Non-MetS (*n* = 151)	MetS (*n* = 152)
Age (yr.)	47 (35–59)	57 (47–66) ^#^	40 (32–51)	57 (48–62) ^#^
Education (yr.)	8 (4–11)	8 (4–11)	11 (4–12)	6 (4–11) ^#^
SBP (mmHg)	118 (113–127)	130 (121–139) ^#^	109 (102–118)	119 (110–133) *^,#^
DBP (mmHg)	71 (64–76)	76 (69–83) *^,#^	67 (62–72)	73 (65–80) *^,#^
Waist circ. (cm)	88 (83–93)	98 (93–106) *^,#^	88 (80–95)	94 (89–102) *^,#^
Fasting glucose (mg/dL)	85 (79–91)	90 (76–101) *^,#^	83 (76–90 )	92 (83–101) *^,#^
HbA1c (%)	5.2 (5.0–5.4)	5.4 (5.2–5.8) *^,#^	5.1 (4.8–5.4)	5.5 (5.2–5.9) *^,#^
HDL (mg/dL)	43 (36–50)	38 (34–42) *^,#^	54 (47–63)	43 (38–48) *^,#^
Triglycerides (mg/dL)	117 (90–145)	189 (139–251) *^,#^	109 (81–135)	169 (130–234) *^,#^
ICV (mm^3^)	1,525,826 (1,455,052–1,650,918)	1,535,378 (1,454,606–1,634,882)	1,399,916 (1,315,294–1,491,247)	1,390,358 (1,316,217–1,466,832)

Data are presented as median (Q1–Q3). * *p* < 0.001 (one sample Wilcoxon signed-rank test) compared to the Joint Interim Statement (JIS) criteria thresholds for metabolic syndrome, which are as follows: systolic blood pressure (SBP) ≥ 130 mmHg, diastolic blood pressure (DBP) ≥ 85 mmHg, fasting glucose ≥100 mg/dL, HDL-c < 40 mg/dL (men) or <50 mg/dL (women), triglycerides ≥150 mg/dL, and waist circumference ≥90 cm (men) or ≥80 cm (women). Glycated hemoglobin (HbA1c) is not a JIS criterion but was included as additional evidence for insulin resistance (reference normal values < 5.7%). ^#^ *p* < 0.001 (Mann–Whitney U test) compared to the Non-MetS group of the same sex. ICV, intracranial volume.

**Table 2:** Regions of interest comparisons in men and women below the 50% age quantile (Q2).

		Men		Women	
		Non-MetS (*n* = 63)	MetS (*n* = 27)	Non-MetS (*n* = 113)	MetS (*n* = 47)
	Age (yr.)	36 (29–42)	38 (33–47)	36 (30–43)	41 (32–47) ***
	Education (yr.)	11 (6–12)	9.5 (7.0–11.0)	11 (6–13)	11 (7–13)
	**ROI**				
	Hippocampus	0.27 (0.26–0.30)	0.27 (0.26–0.28)	0.27 (0.27–0.30)	0.27 (0.36–0.29)
	Entorhinal	0.24 (0.20–0.27)	0.24 (0.21–0.27)	0.24 (0.22–0.26)	0.24 (0.22–0.27)
Right	Middle temporal gyrus	0.56 (0.53–0.60)	0.57 (0.51–0.60)	0.58 (0.52–0.59)	0.55 (0.50–0.59)
	Precuneus	0.35 (0.32–0.37)	0.36 (0.34–0.38)	0.34 (0.31–0.36)	0.34 (0.32–0.36)
	Amygdala	0.102 (0.092–0.108)	0.100 (0.092–0.103)	0.100 (0.086–0.100)	0.100 (0.090–0.106)
	Hippocampus	0.27 (0.25–0.28)	0.27 (0.25–0.28)	0.28 (0.27–0.30)	0.27 (0.26–0.28) **
	Entorhinal	0.21 (0.19–0.24)	0.22 (0.18–0.23)	0.22 (0.19–0.25)	0.22 (0.19–0.26)
Left	Middle temporal gyrus	0.52 (0.49–0.56)	0.51 (0.48–0.57)	0.51 (0.48–0.57)	0.48 (0.43–0.53) *
	Precuneus	0.35 (0.31–0.38)	0.36 (0.33–0.38	0.34 (0.31–0.38)	0.34 (0.32–0.39)
	Amygdala	0.094 (0.089–0.100)	0.095 (0.088–0.100)	0.091 (0.085–0.100)	0.092 (0.087–0.101)

Regions of interest (ROI) as % of the intracranial volume. Median (Q1–Q3). *** *p* < 0.001 (Mann–Whitney U test), ** *p* = 0.02, and * *p* = 0.05 (quantile regression analysis) compared to the Non-MetS group of women.

**Table 3:** Regions of interest comparisons in men and women above the 50% age quantile (Q2).

		Men		Women	
		Non-MetS (*n* = 52)	MetS (*n* = 55)	Non-MetS (*n* = 38)	MetS (*n* = 105)
	Age (yr.)	61 (55–68)	62 (56–69)	58 (55–65)	60 (56–65)
	Education (yr.)	4 (4–10)	8 (4–11) ^#^	5 (4–11)	4 (4–8)
	**ROI**				
	Hippocampus	0.26 (0.24–0.28)	0.27 (0.24–0.28)	0.27 (0.25–0.29)	0.28 (0.26–0.29)
	Entorhinal	0.25 (0.23–0.28)	0.24 (0.21–0.27)	0.25 (0.22–0.26)	0.24 (0.22–0.28)
Right	Middle temporal gyrus	0.52 (0.48–0.56)	0.53 (0.49–0.55)	0.53 (0.47–0.56)	0.52 (0.48–0.56)
	Precuneus	0.33 (0.31–0.35)	0.35 (0.30–0.37)	0.33 (0.29–0.36)	0.35 (0.32–0.37)
	Amygdala	0.095 (0.086–0.106)	0.095 (0.087–0.105)	0.090 (0.083–0.101)	0.098 (0.089–0.105) *
	Hippocampus	0.26 (0.24–0.28)	0.26 (0.23–0.28)	0.26 (0.25–0.29)	0.27 (0.25–0.29)
	Entorhinal	0.22 (0.19–0.25)	0.22 (0.18–0.25)	0.22 (0.20–0.24)	0.22 (0.20–0.25)
Left	Middle temporal gyrus	0.47 (0.42–0.52)	0.49 (0.46–0.53)	0.47 (0.44–0.52)	0.47 (0.43–0.52)
	Precuneus	0.32 (0.29–0.35)	0.33 (0.30–0.37)	0.33 (0.30–0.36)	0.34 (0.30–0.37)
	Amygdala	0.090 (0.082–0.098)	0.089 (0.084–0.099)	0.092 (0.084–0.099)	0.092 (0.084–0.098)

Regions of interest as % of the intracranial volume. Median (Q1–Q3). Quantile regression analysis: * *p* < 0.001 compared to the Non-MetS group of women (right amygdala). Mann–Whitney U test: ^#^ *p* < 0.001 compared to the Non-MetS group of men (education).

**Table 4:** Quantile regression parameter estimates (Q2) for selected ROI in women.

Dependent Variable (ROI/ICV)	Subgroup (Age)	Coefficient (βMetS)	Standard Error	*p*-Value	Interpreted Effect
Left Hippocampus	Younger Women (≤Q2)	0.0001	4.41 × 10^−5^	0.02	Non-MetS > MetS (Reduced Volume in MetS)
Left Middle Temporal Gyrus	Younger Women (≤Q2)	0.0003	0.0002	0.05	Non-MetS > MetS (Reduced Volume in MetS, Trend)
Right Amygdala	Older Women (>Q2)	−9.41 × 10^−5^	2.78 × 10^−5^	<0.001	MetS > Non-MetS (Increased Volume in MetS)

ROI: cerebral cortex region of interest. ICV: intracranial volume. Q2: 0.5 quantile (median).

## Data Availability

The database analyzed in the current study is the property of the Baependi Heart Study project. This project conducts various research initiatives continuously and over time. Therefore, to prevent overlapping with ongoing or similar research endeavors, researchers interested in accessing the data and biomaterial must submit a detailed proposal outlining their specific needs and research objectives. Researchers can apply for data and biomaterial by submitting a proposal to the Project Coordinator, Dr. Alexandre C. Pereira (alexandre.pereira@incor.usp.br).
